# Upconversion Photoluminescence to Monitor Local Heat Release During Femtosecond Direct Laser Writing of Bioinks In Situ

**DOI:** 10.1002/smtd.202502130

**Published:** 2026-02-08

**Authors:** Amirbahador Zeynali, Giuseppe Chirico, Michael Heymann

**Affiliations:** ^1^ IBBS Institute for Biomaterials and Biomolecular Systems University of Stuttgart Stuttgart Germany; ^2^ Department of Physics University of Milano‐Bicocca Milano Italy

**Keywords:** bioprinting, femtosecond direct laser writing, in situ thermometry, stereolithography, two‐photon polymerization, upconversion nanothermometry

## Abstract

Exothermic photopolymerization releases heat into the sample environment. Using NaYF_4_:Yb^3+^/Er^3+^ upconversion nanoparticle (UCNP) photoluminescence and a colinear lithography and thermometry laser configuration, we monitor thermal signatures in the focal spot during femtosecond direct laser writing in real time. A statistical short‐pass filtering is introduced to reduce the standard error in temperature calibration compared to conventional Gaussian deconvolution. Thermometry performance of our set‐up achieved a relative sensitivity of 0.89–1.58% K^−1^ and a measurement uncertainty of 0.2–0.4 K for 2 Hz sample rates. With this, the effect of scan speed, laser power, and photoinitiator concentration on accompanying local heating could be followed. Nonlinearities and thermal runaway effects with transient temperature spikes above 120–140°C demonstrate the need for a stringent reduction of the thermal burden when writing aqueous bioinks for biomedical applications. Physiological conditions were maintained only for fast 20 µm/s scan speeds, which limited temperature quenches to not exceed physiological temperatures. This paves the way to improve process control and to optimize for laser‐assisted bioprinting and other related technologies.

## Graphical abstract

Upconversion nanoparticle photoluminescence was used to quantify heat burden during femtosecond direct laser writing of bioinks in real time. Transient temperature spikes above 120–140°C demonstrate the need for a stringent process optimization, and fast 20 µm/s scan speeds were established to maintain physiological conditions.

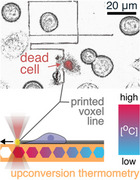

## 1 Introduction

The combination of high peak intensities with comparably low average power of femtosecond (fs) pulses spaced a few nano‐ or even microseconds apart has become invaluable for multiphoton microscopy, submicron material 3D processing, and microsurgery [1, 2, 3, 4, 5, 6]. For these, near‐infrared (NIR) light is deposited deeply into a photo‐responsive material with high spatial and temporal precision via two‐ and three‐photon excitation. This is because the almost instantaneous absorption of multiple photons scales nonlinearly with the incident light intensity. Excitation of a fluorophore or photoinitiator [7, 8] is limited to the focal spot, where the critical excitation threshold is only exceeded for the brief duration of the femtosecond pulse. Typically, pulse peak intensities of order 0.1 TW cm^−2^ are required to induce nonlinear absorption processes [9, 10]. In a biomedical context, this approach afforded deep tissue imaging while incurring lower volumetric photodamage compared to out‐of‐focus photobleaching incurred from one‐photon imaging, which remains a concern in live cell and in vivo animal imaging [11], such as in neurobiology [12]. Multiphoton polymerization, in turn, can realize complex 3D objects from synthetic photoresists [13], or bioinks suited for cell culture [14, 15, 16], that were also used intravitally already [17].

To minimize light induced damage, both instantaneous and integrated excitation power densities should be optimized. Reaction initiation itself, leading to “cold” polymerization, is a nonlinear composite of laser power, pulse duration, and repetition rates, focal spot size, as well as scan speeds and spacing [10]. In addition, an exothermic polymerization reaction can enter a positive feedback loop, where heat from the exothermic reaction increases temperature to, in turn, accelerate the polymerization rate, which further increases heat generation [10, 18, 19, 20]. As a result, different voxel curing temperatures engrain strain gradients across the final object, which, for instances is a particular concern in multiphoton printed microoptics, where even minute shape deviations from the desired optical surface markedly deteriorate performance [21, 22]. From a biosafety perspective, power densities should not exceed the critical tissue damage threshold. Photothermal damage may result from direct excitation, followed by non‐radiative relaxation that transfers energy to the lattice, generating heat, or indirectly through exothermic heat incurred from the desired reaction or undesired side‐reactions [10, 23, 24, 25, 26, 27]. Temperatures above ∼43°C can amplify reactive radicals and oxygen species, rupture membranes, induce irreversible protein unfolding and aggregation, impair proteostasis and activate caspases [28, 29, 30], to ultimately prompt cell death via necrosis or apoptosis [10, 24]. In neuronal cells, localized heating has been linked to calcium dysregulation [31] and mitochondrial dysfunction, accelerating apoptotic pathways [32]. Repeated or sustained thermal injury at the tissue or systemic level confers chronic inflammation characterized by cytokine release, tissue remodeling, and fibrotic changes [33], which has been implicated in carcinogenesis, particularly in heat‐exposed organs such as the skin, liver, and gastrointestinal tract [34].

Accessing local temperature fluctuations around the focal spot during multiphoton excitation in real‐time will be invaluable to safeguard high‐resolution fs‐laser applications. Prior efforts estimated local temperatures under femtosecond laser exposure [35, 36], but in relying on indirect models and simulations have fallen short of capturing full experimental temperature dynamics at the microscale. A broad range of luminescent and non‐luminescent nanoscale thermometers have been developed for use in electronics, photonics, microfluidics, and biomedicine [37, 38, 39, 40]. These include temperature‐responsive organic dyes [41], fluorescent proteins [42], polymer‐based sensors [43], and nanomaterials doped with transition metals [44, 45] or trivalent lanthanide ions [46, 47]. In the nanoscale regime, quantum dots [48], carbon dots [49], gold nanoclusters [50], nanodiamonds [51], and both downshifting and upconverting phosphors [52, 53, 54, 55] have excelled as luminescent thermometers. Especially, lanthanide‐doped upconversion nanoparticles (UCNPs) are distinguished by their ability to convert low‐energy excitation (typically in the near‐infrared) into higher‐energy emission, through anti‐Stokes processes. This allows for deeper light penetration with minimal autofluorescence and scattering, making UCNPs especially suitable for biological applications [56, 57]. UCNPs contain a sensitizer ion (e.g., YB^3+^) to absorb NIR photons and transfer energy to an activator ion (e.g., Er^3+^), which subsequently emits light. β‐phase sodium yttrium fluoride (β–NaYF_4_) is widely recognized as the most efficient host matrix for lanthanide doping. Its low phonon energy (∼350 cm^−1^) significantly suppresses non‐radiative relaxation pathways, thereby enhancing radiative emission for bright upconversion luminescence under near‐infrared excitation [58]. Various UCNP host materials have been tailored for high photostability in bioimaging [59], optimized phonon energy in thermal sensing [60, 61], high refractive index contrast in photonic integrated chips [62, 63], and efficient charge transfer in photocatalysis [64, 65].

Real‐time in situ thermometry is already established for investigating two‐photon excitation processes, yet its application in biofabrication, particularly with biologically derived photoresists, remains unexplored. Embedded within the photoresist, UCNPs helped to quantify local heat spikes as high as ∼300°C when writing pentaerythritol triacrylate [66] or SZ2080 [67]. Such heat spikes would be catastrophic for protein‐based scaffolds and, by extension, for nearby cells, since they can trigger photoresist degradation [68]. Notably, Yb^3^/Er^3+^ co‐doped glass nanothermometers were adopted for multiphoton optogenetic imaging to help limit induced excess heat to a few degrees [18]. Generally, UCNPs exhibit a temperature‐dependent emissivity, with their luminescence decreasing for higher temperatures [69, 70], as non‐radiative relaxation becomes dominant. The comparably low physiological temperatures of these biological experiments will hence require low excitation powers and sensor particle concentrations to efficiently monitor local temperature.

**FIGURE 1:**
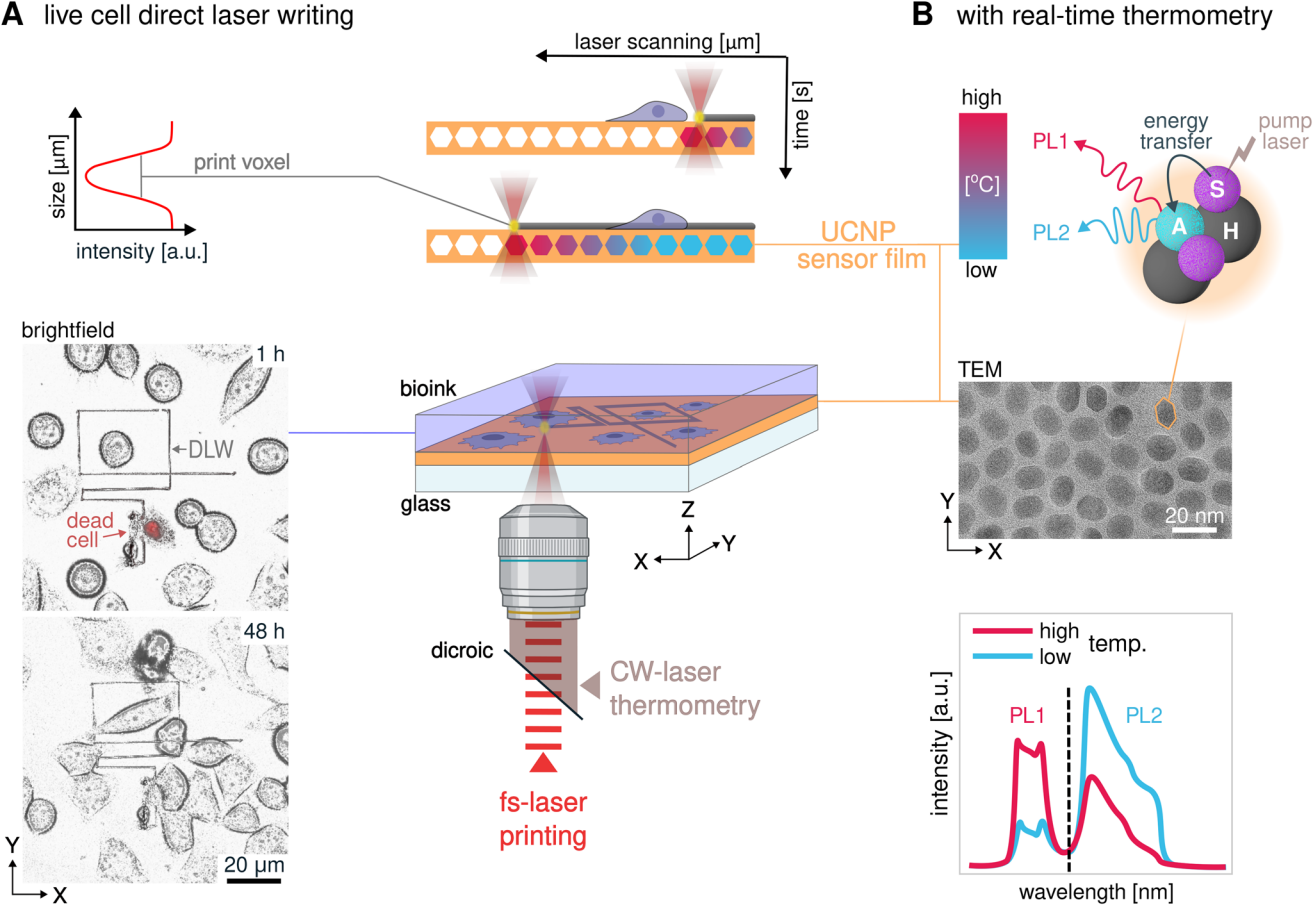
In situ nanothermometry to quantify excess heat during multiphoton 3D bioprinting. (A) Measurement concept for direct laser writing in the presence of living cells with simultaneous thermometry via an UCNP sensor film substrate (top left panel). HeLa cell culture with propidium iodide dynamic cell viability assay 1 and 48 h after bioprinting. While continuous proliferation demonstrates general biocompatibility of the process, cells directly intersected by the laser die off quickly (bottom left). (B) Transmission electron micrograph of nanoparticle sensor film. Host, H, sensitizer, S, and activator, A, ion interaction result in upconversion nanoparticle photoluminescence, PL. Thermally sensitive energy levels can be quantified via intensity ratios of the corresponding emission ranges (bottom right panel).

Here, we set out to monitor heat generation and dissipation in real‐time during fs‐direct laser writing in situ (Figure 1A). For this, a photoluminescence thermometer composed of ∼20 nm Yb^3+^/Er^3+^ co‐doped UCNPs was integrated into the writing substrate (Figure 1B). UCNPs are photothermally excited with a continuous wave laser co‐aligned with the pulsed fs‐lithography laser. A spectrograph can then record emitted luminescence spectra every second in situ. From these, temperature quenches at the focal spot, ranging from room temperature up to 145°C could be quantified. Accuracy in ratiometric photoluminescence thermometry hinges on accurate corrections for background and non‐thermal intruding transitions [40, 71, 72]. We introduce a filtering‐based approach to refine ratiometric analysis, enabling more sensitive and accurate extraction of the activator ion energy gap (Δ*E*) than achievable with spectral deconvolution [73]. We apply this set‐up to track excess heat accumulation when writing bovine serum albumin (BSA) protein hydrogel microstructures. Even brief exposures of the fs‐laser could induce acute cytotoxicity when excess heat accumulated at the polymerization voxel raised the temperature to well over 100°C, which was well above the viability limit of HeLa cells. Spot‐wise writing strategies and reduced photoinitiator concentrations could mitigate these extreme, yet highly localized temperatures. We expect this technique to help access and reduce laser induced damage in engineered materials, especially in biologically active environments.

## 2 Results and Discussion

### 2.1 Experimental Configuration for Real‐time Nanothermometry during Direct Laser Writing

**FIGURE 2:**
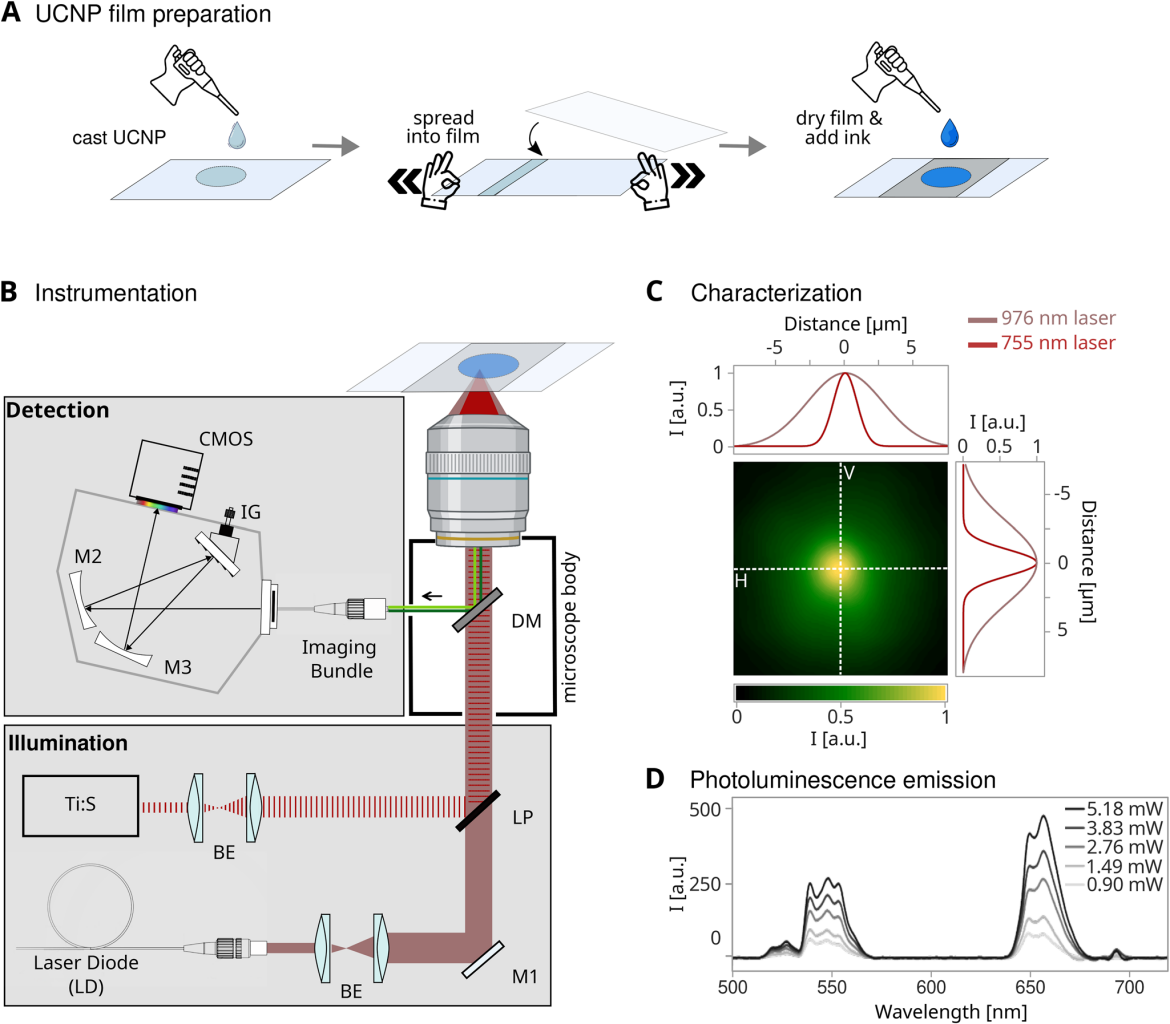
Real‐time UCNP‐thermometry assay configuration. (A) Schematic drop‐casting workflow to prepare a thin UCNP film on a microscope slide, overlaid with BSA‐MB‐bio‐ink for in situ thermometry during direct laser writing. (B) Optical setup: a 755 nm fs‐pulsed Ti: sapphire laser and a 976 nm CW laser from a fiber‐bragg‐grating laser diode, FBG‐LD, are co‐aligned using a long pass, LP, dichroic and focused through a 60x, 0.85 NA objective. Upconverted Er^3+^ emission is collected via a spectrograph integrated into the setup with a beam expander, BE, dichroic mirror, DM, folding mirrors, M1‐3, and an integrative grating, IG. (C) Superimposed lateral beam profiles show a tight focal spot for the 755 nm lithography beam, with a full‐width at half‐maximum, FWHM at the focal plane of 1.8 and 2.0 µm in horizontal and vertical direction. The 976 nm thermometry beam was aligned through the same focusing module to a FWHM of 5.7 and 6.1 µm, respectively at the same focal plane, with both peaks spaced just about 10 nanometers apart. (D) Representative Er^3+^ emission spectra from NaYF_4_:Yb^3+^/Er^3+^ UCNPs under varying excitation conditions.

To validate temperature readout and beam co‐registration, NaYF_4_:Yb^3+^/Er^3+^ UCNPs were first characterized for their size (Figure S1 and Section S1) and then immobilized by drop‐casting onto a glass slide and air‐dried to prepare a thin film (Figure 2A). The resulting UCNP layer displayed uniform photoluminescence across the ∼120 × 80 µm field of view under 976 nm continuous‐wave excitation. Subsequent BSA and methylene blue (MB) bio‐ink writing proceeded directly on this UCNP surface.

The 976 nm thermometry excitation laser was colinearly aligned into a custom‐built two‐photon bioprinter [74], with a ∼200 fs, 80 MHz Ti:Sapphire laser producing peak intensities of ~0.4 TW cm^−2^ when focused into the sample through a dry 60x, NA = 0.85 objective lens (Figure 2B; Section S2). Note, two‐photon polymerization or stereolithography and femtosecond direct laser writing (fs‐DLW) have been coined and are commonly used interchangeably. For consistency, we refer to the latter in this manuscript. Both excitation foci were offset laterally by no more than ∼10 nm in the focal plane and exhibited a full‐width at half‐maximum of 5.7 ± 0.3 µm × 6.1 ± 0.8 µm for the thermometry laser and 1.8 ± 0.3 µm × 2.0 ± 0.8 µm for the lithography laser, as confirmed by Gaussian fits of the respective beam profiles (Figure 2C). In a typical Gaussian laser beam, intensity is described in terms of power per unit area (W · cm^−2^), corresponding to the beams cross‐sectional area. This dual colinear beam configuration with different beam waists presents different tradeoffs. First, a desirable tight writing spot is combined with a larger detection window to overall improve signal‐to‐noise levels, as more UCNP signal is integrated. Second, too large a thermometry spot in turn would wrongly dilute the local heat signal quantified at the lithography focus. The instantaneous peak temperature at the absolute center of the 1.8 µm voxel should hence exceed the reported average. In principle, heat diffuses about 3 µm in water within 100 µs, which is significantly faster than the sampling rate of our detector of 500 ms. Also, note that the effective interaction volume along the propagation axis becomes critical, producing an intensity distribution that extends into the third dimension [75, 76].

NaYF_4_:Yb^3+^/Er^3+^ particles have a weak absorption tail in the range of 780–800 nm, a spectral window popular in two‐photon lithography [40, 71]. To exclude undesired low‐level upconversion from parasitic excitations, the lithography laser was tuned to 755 nm with 50–100 mW average power. BSA–MB bioink could easily be written under this 755 nm lithography laser excitation. Also, no UCNP emission was observed, suggesting sufficient spectral separation from the higher primary Yb^3+^ excitation band and thus negligible Er^3+^ upconversion emission.

The NaYF_4_:Yb^3+^/Er^3+^ thin‐film temperature sensor substrate performance was then calibrated for 976 nm CW diode laser excitation powers between 0.9 and 5.18 mW (Figure 2D). A Peltier stage maintained a constant temperature for accurate mapping of emitted luminescence intensity ratios to absolute temperature. Distinct Er^3+^ emission peaks centered at ∼525 nm (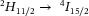), ∼545 nm (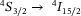), and ∼655 nm (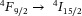) were observed, with overall intensities strongly correlating with excitation power, as confirmed from their localization in CIE 1931 chromaticity coordinates (Figure S2 and Section S3).

### 2.2 Intruder Band Correction Improves NaYF_4_:Yb^3+^/Er^3+^ UCNP Thermometry Accuracy

**FIGURE 3:**
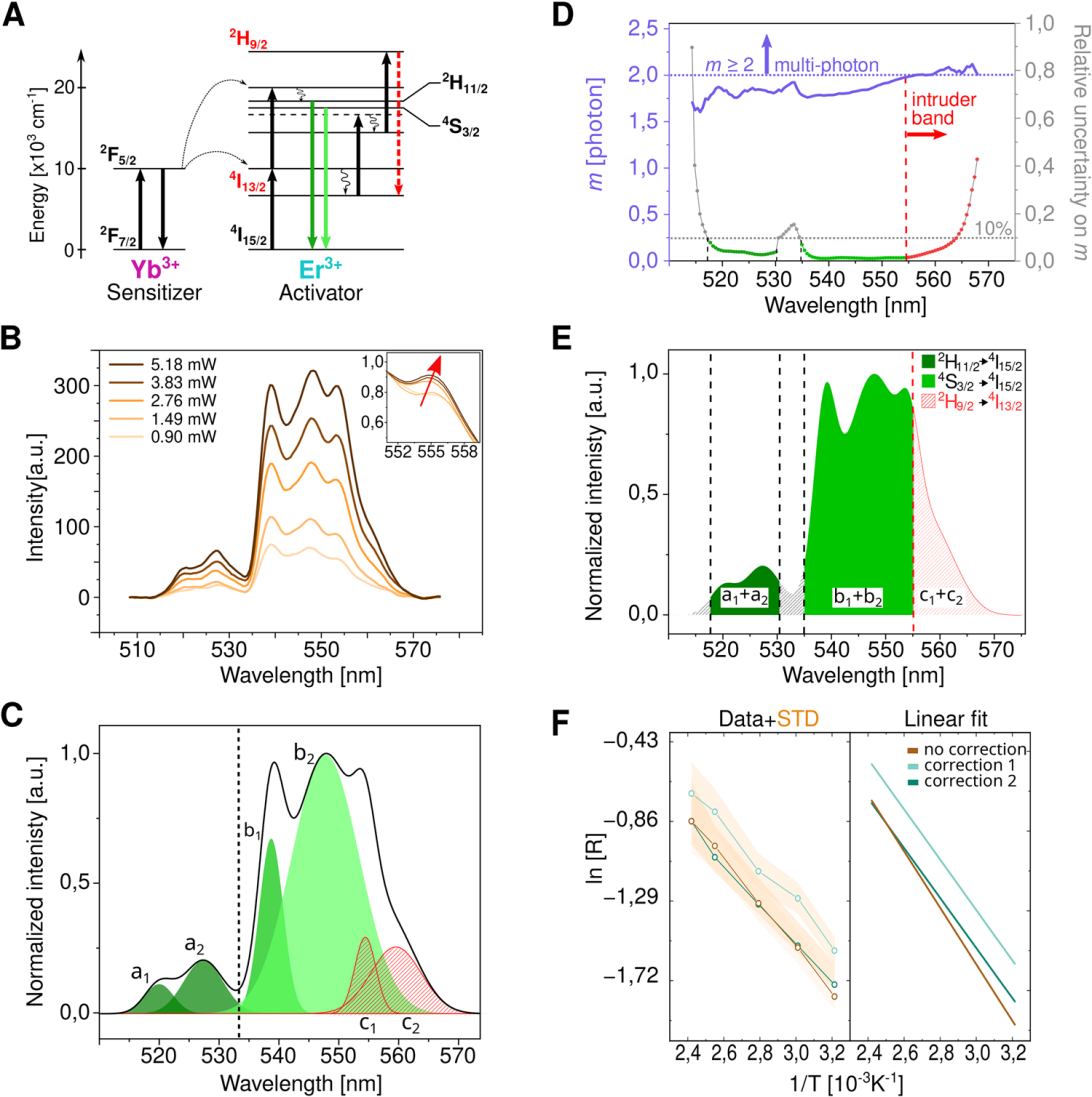
Spectral correction to calibrate NaYF_4_:Yb^3+^/Er^3+^ UCNP photoluminescence. (A) Simplified energy diagram of *Yb* ^3 +^ /*Er* ^3 +^ upconversion, highlighting thermally coupled ^2^ *H*_11/2_ and ^4^ *S*_3/2_ energy states, whose emission intensity ratio enables temperature quantification. An intruder band ^2^ *H*_9/2_ → ^4^ *I*_13/2_ (red dashed line) contaminates the emission spectrum at higher excitation energies and should be corrected. (B) UCNP photoluminescence spectra for increasing 976 nm excitation intensities. Normalization reveals an excitation power‐dependent artefact above 550 nm (inset). (C) Multi‐Gaussian spectral deconvolution identifies and excludes non‐thermal components highlighted in red from ratiometric analysis. (D) Alternatively, a power‐law analysis (*I* ∝ *P^m^*) across the emission range can help identify multiphoton transitions with *m* > 2. In combination with spectral filtering to exclude high uncertainty emission regions (>10%) the range of 514–531 and 534–554 nm was identified as thermal emission window suitable for thermometric quantification. (E) Resulting corrected spectra for the thermometry measurement window indentified in (D). (F) The experimental Boltzmann ratio and relative standard deviation (left panel) and linear calibration fits (right panel). Accurate retrieval of the Er^3+^ energy gap (ΔE) between the thermally coupled transitions was achieved only after applying the second correction (D,E), highlighting its effectiveness in isolating reliable temperature‐sensitive emission components.

UCNP‐based thermometry easily overestimates the energy gap (Δ*E*) between thermally coupled 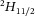 and 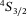 Stark levels for Er^3+^ ions, when spectral overlaps and non‐thermal emission components remain uncorrected (Figure 3A). Excitation at 976 nm drives a stepwise two‐photon process in the Yb^3+^/Er^3+^ system, with Yb^3+^ ions sequentially transferring energy to Er^3+^ ions. This populates multiple intermediate excited states that give rise to two primary emission bands associated with thermally coupled transitions 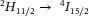 and 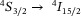 centered at approximately 525 nm (*I*_525_) and 545 nm (*I*_545_) [40, 71]. For UCNPs co‐doped NaYF_4_, the expected energy separation between these Stark sublevels is approximately 700–760 cm^−1^ [77, 78, 79]. The temperature‐dependent intensity ratio R is typically calibrated via a Boltzmann relationship, defined by the integrated emission intensities of two thermally coupled Er^3^^+^ transitions, with maxima around 525 and 545 nm [40]:

(1) $$R=\frac{I_{525}}{I_{545}}=c\;e^{-\frac{\Delta E}{k_{B}T}}$$

In Equation (1) Δ*E* is the energy gap between 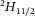 and 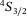, *c* is a degeneracy‐related constant, and *k_B_* the Boltzmann constant. At constant temperature, *T*, when the 976 nm excitation power exceeded 2.76 mW, the spectral region between ∼550 and 570 nm revealed additional emission components originating, for example, from transitions such as 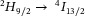 (Figure 3B) [37]. These higher‐order features are considered independent of the thermometric transitions at *I*_525_ and *I*_545_ [40].

Nonlinear spectral distortions and deviations from ideal Boltzmann behavior within the 533–570 nm range, belonging to the 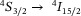 transition (Figure 3C) are analyzed with two complementary correction strategies, aimed to exclude emission components that fail to conform to the expected photophysical characteristics of thermally coupled Er^3^^+^ transitions. The Er^3^^+^ contaminating criterion was identified by extending the conventional power‐law approximation between emission intensity, *I*, which is commonly modeled as a function of excitation power, *P*, [80, 81]:

(2) $$I\propto P^{m}$$

to a statistically more robust analysis, as [75, 76].

(3) $$I=\frac{A\left(\lambda \right)P^{n}}{1+B\left(\lambda \right)P}\;$$

which simplifies for high powers, *B*(λ)*P* ≫ 1, to

(4) $$I≃A^{\prime }\left(\lambda \right)\;P^{n-1}=A^{\prime }\;\left(\lambda \right)P^{m}$$

with *m*  =  *n* − 1 and $A^{\prime }\;\left(\lambda \right)=\frac{A(\lambda )}{B(\lambda )}$. The power dependence for each component was then evaluated, with those exhibiting a multiphoton order, *m* > 2, identified as intruder bands. In fact, for *m* > 2,  at least 3 photons are involved in priming the emission, confirming the involvement of high energy levels (Figure 3A). In a first correction strategy, a conventional multi‐Gaussian spectral deconvolution (Correction 1) allowed to assign overlapping emission bands, but was excluded from further analysis (Figure 3C; Figure S3 and Section S4) [40, 73]. In a different approach, we quantify the fitting uncertainties of the power‐law exponent *m* across the complete emission spectrum (Figure 3D; Section S4). Spectral regions exhibiting a relative uncertainty exceeding 10% were statistically segmented and excluded from ratiometric thermometric analysis. This constitutes an alternative correction method (Correction 2) to remove contributions from the identified intruder range (554–568 nm) in the thermometry signal. Considering both the power‐law exponent and the relative measurement uncertainty, we defined suitable spectral integration windows for the thermally coupled Er^3^^+^ transitions as 514–531 and 534–554 nm. Emissions above 554 nm (Figure 3E) exhibit higher‐order multiphoton behavior and increased uncertainty, consistent with non‐thermal intruder contributions. Correction 2 can be conceptualized as a mathematically rigorous short pass filtering at a spectral cutoff at 554 nm to isolate the optically and thermodynamically coherent transitions. This allows to bypass Gaussian deconvolution entirely.

Temperature calibration was based on five experimental spectra in the range of 35–150°C by plotting ln(R) against the inverse temperature (1/*T*) (Table 1 and Figure 3F):

(5) $$\ln\left(R\right)=-\frac{\Delta E}{k_{B}}\;\frac{1}{T}+\ln\left(c\right)$$

**TABLE 1:** Boltzmann calibration parameters derived from raw and corrected UCNP emission spectra. The standard deviation represents the standard error of the fitted ΔE value obtained from linear regression of the Boltzmann plot, reflecting the statistical uncertainty associated with the calibration fit.

Approach	|Δ*E*/*k_B_* | (slope)±STD	Intercept	|Δ*E*| [cm^−1^]
No correction	1203.36 ± 22.59	2.02	836.34
Gaussian deconvolution (Correction 1)	1072.29 ± 72.73	1.86	745.94
Statistical short pass filtering (Correction 2)	1064.77 ± 29.12	1.67	740.02

Both corrections achieved good agreement with the expected theoretical energy gap (Δ*E*) between the thermally coupled states of NaYF_4_:Yb^3+^/Er^3+^ nanoparticles [60, 82]. Intriguingly, the statistical short pass filtering exhibited a strongly decreased standard error, compared to the Gaussian deconvolution, and an excellent agreement with the expected energy separation between the Stark sublevels of ≃700–760 cm^−1^ [77, 78, 79] (Section S5).

Precision and responsiveness of luminescent thermometers are commonly evaluated as the relative sensitivity, *S_r_*, and the temperature measurement uncertainty, δ*T*, [40]:

(6) $$S_{r}=\frac{1}{R}\;\left|\frac{\partial R}{\partial T}\right|≃\frac{\Delta E}{k_{B}T^{2}}$$

(7) $$\delta T=\frac{1}{S_{r}}\;\frac{\delta R}{R}$$

The uncertainty in the intensity ratio δR was obtained from independent integration errors of the two emission bands, computed as:

(8) $$\delta R=\sqrt{{\left(\frac{\delta I_{525}}{I_{525}}\right)}^{2}+{\left(\frac{\delta I_{545}}{I_{545}}\right)}^{2}}\;\left(R\right)$$

where δ*I*_525_ and δ*I*_545_ represent the standard error of the numerically integrated, background‐corrected spectral windows. Note, both *S_r_* and δ*T* are temperature‐dependent. UCNP sensor performance can hence vary across different temperature regimes, particularly between ambient and physiological temperatures.

The thermometry performance of our UCNP substrate showed a relative sensitivity of 0.89−1.58% K^−1^ and a measurement uncertainty of 0.2–0.4 K (Table 2), which agrees well with previously reported configurations that achieved performances of 0.15−1.7% K^−1^ and 0.29−3.3 K respectively [60]. Importantly, this demonstrates that the precision and sensitivity of the UCNP sensor here can be preserved even under the optically and thermally demanding conditions of in situ fs‐DLW. Over all, the proposed statistical short pass filtering (Correction 2) improved the accuracy of ratiometric temperature sensing by minimizing the spectral overlap between thermally sensitive and non‐sensitive bands. We consider this reduction in oversampling error to be especially beneficial when mapping dynamic temperature responses, such as the highly nonlinear spatiotemporal thermal effects of fs‐DLW.

**TABLE 2:** UCNP slide performance, including relative sensitivity, S_r_, and temperature measurement uncertainty, δT. The standard deviation of the ratio R values, δR, over the range of applied temperatures, along with the mean value of the integrated areas ratio, R, was used to calculate the measurement uncertainties.

Temperature [°C; K]	*S_r_* [%K^−^^1^]	*R*	$\frac{\delta R}{\bar{R}}$	δ*T* [K]
38; 311.15	1.58	0.17	0.35	0.2
59; 332.15	1.39	0.21		0.2
85; 358.15	1.19	0.27		0.3
119; 392.15	0.99	0.37		0.3
140; 413.15	0.89	0.42		0.4

### 2.3 Polymerization Process Thermometry of a Single Voxel Exposure

Equipped with this sensitive and accurate thermometry platform, we set out to quantify excess heat production during bioink fs‐DLW. As a first test, the 755 nm femtosecond lithography laser was simply focused on a fixed position into the bioink just above the UCNP film, while controlling the 5 s exposure of a single voxel with a mechanical shutter. UCNP luminescence was observed under 976 nm continuous‐wave laser excitation with a 5.2 mW average power, and emission spectra were recorded every 0.5 s (1 frame/0.5 s) for real‐time in situ thermometry.

**FIGURE 4:**
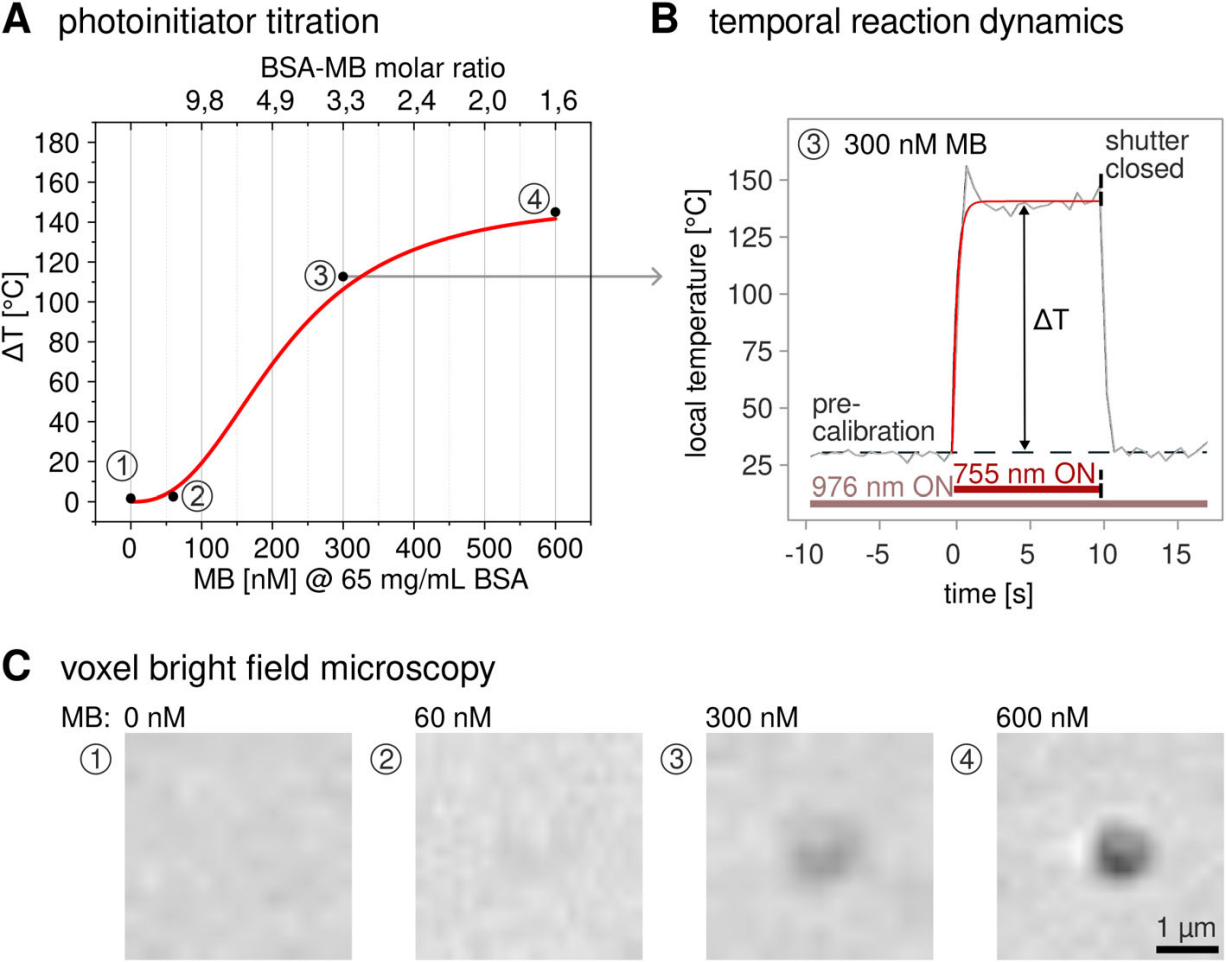
Real‐time thermometry of a single voxel exposure. (A) stationary 60 mW, 755 nm fs‐lithography laser exposure of 10 s was used to crosslink a 65 mg/ml BSA bioink with varying concentrations of MB. While no noticeable heat accumulation could be observed in the absence of the photoinitiator, a temperature plateau of up to 140°C was reached in the limit of high concentrations for 600 nm MB, with the Δ*T* being the difference between pre‐calibrated background temperature and the steady state peak. (B) For instances, at 300 nM MB voxel temperatures reached Δ*T* = 140.5°C −  27.8°C ≃  112.6°C. (C) Corresponding voxel morphologies evolved from no noticeable gelation in the absence of MB, to incipient nuclei at 60 nm, faint gel for 300 nm to pronounced gelation at 600 nM MB.

We chose a 65 mg/ml (977 µM) BSA and methylene blue (MB) as the proteinatious bioink due to its simplicity and widespread use [6, 74, 83]. Each exposure of the 755 nm femtosecond lithography laser placed 60 mW average power into a voxel of 0.35 µm width and 2 µm height. BSA inks with no or low concentration of ≃60 nm MB accumulated only small quantities of heat, resulting in temperature increases of Δ*T*  =  1.5 ± 0.3°C and Δ*T*  =  2.6 ± 0.3°C above lab background temperature *T*_0_ ≃ 27.8°C (Figure 4). Here, no or at best a very limited sample densification could be observed, suggesting only limited cross‐linking within the exposed voxel. A concentration of 60 nM corresponds to approximately one MB molecule per 16 BSA molecules, indicating a relatively sparse distribution of photoinitiators within the protein matrix. Hence, even with activation of all MB molecules, the local density of reactive species remained insufficient to cross‐link the entire matrix. Moreover, in this low‐concentration regime, a significant portion of the absorbed excitation energy is more likely to dissipate through non‐radiative decay, resulting in localized heat rather than contributing to productive photochemistry. This highlights a critical trade‐off: while lower photoinitiator concentrations minimize photothermal damage and preserve protein structure, they also reduce cross‐linking efficiency. Conversely, increasing the concentration may enhance network formation but at the cost of elevated local heating and potential protein denaturation. In fact, an increase of MB concentration to 300 or 600 nM, corresponding to roughly one MB per 3 or 1.5 BSA molecules. Corresponding steady state temperature increased to well above the water boiling point with Δ*T*  =  112.6 ± 0.3°C and Δ*T*  =  144.9 ± 0.3°C, respectively [84] (Figure 4A,B). These temperature increases, achieved within just 3 s of irradiation, are consistent with a photothermal runaway effect [85, 86]. We reason that initial heating promotes faster polymerization via the exothermic reaction, which in turn generates additional heat, creating a positive feedback loop that rapidly elevates local temperatures. Such behavior underscores the importance of controlling photoinitiator concentration to balance cross‐linking efficiency with thermal safety, particularly in protein‐based photoresists [68].

Distinct spots were fabricated that could easily be discerned in bright field microscopy (Figure 4C). At low photoinitiator concentrations, cross‐linking is limited to isolated nucleation events with faint or incomplete features forming only at higher laser intensities. As the MB concentration increases, a smoother and more continuous polymer matrix develops, culminating in full 3D voxel polymerization. Cross‐linking BSA into a hydrogel throughout a focal volume requires critical percolation clusters to form. In principle, this could proceed via chemical cross‐linking downstream from the photoinitiation. Alternatively, a heat induced phase transition of (irreversible) BSA protein unfolding and aggregation can be imagined to proceed after a critical temperature has been surpassed locally. We note that the photothermal response exhibits a pronounced nonlinear increase and ultimately reaches a plateau (Figure 4A), suggesting a limiting process to eventually become dominant. Irrespective of such mechanistic considerations, we sought to avoid non‐physiological conditions and hence looked for more gentle writing schemes.

### 2.4 Thermometry of Sequentially Written Voxel Arrays

**FIGURE 5:**
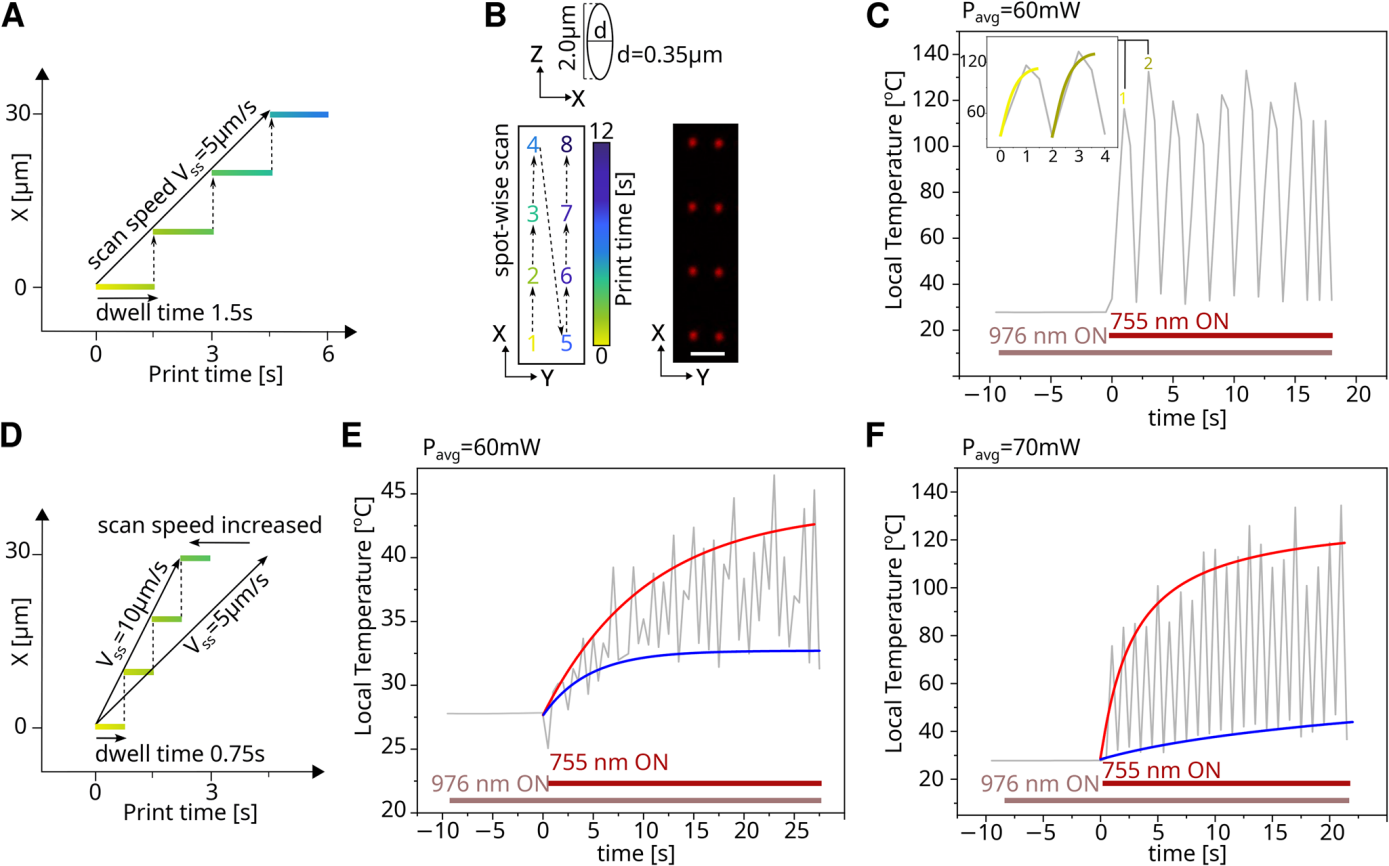
Real‐time thermometry of voxel array writing with 10 µm spacing. (A) Schematic of spot‐wise laser voxel writing with exposure dwell times of 1.5 s, intermitted by fast stage translations to a new voxel position 10 µm away for an effective 5 µm/s scan speed. (B) Theoretical voxel sizes were 0.35 µm in lateral (ΔX) and 2 µm longitudinal (ΔZ) dimensions (top). The programmed voxel raster in the XY plane (bottom left) and resulting voxel arrays (bottom right) were confirmed in confocal microscopy. Scale bar = 5 µm. (C) Sequential voxel writing with 1.5 s exposure dwell times at 60 mW laser power followed a pulsatile heating profile. Exponential fits of the first two exposures (inset) suggest peak temperatures of ∼112°C above background. (D) Shorter dwell times of 0.75 s increase the effective scan speed to 10 µm/s. (E) The correspondingly lower integrated dose for constant lithography laser powers of 60 mW attenuated heating peaks to only ∼17°C (red exponential fit). Local cooling minima in turn equilibrated to a higher value of about ∼5°C above background, consistent with reduced diffusive heat dissipation for the faster scans (blue exponential fit). (F) A slightly increased laser power of 70 mW induced pronounced temperature spikes above 100°C, implicating both laser power and the scan speed to independently govern nonlinear heat accumulation during fs‐direct laser writing.

To quantify proximity effects between neighboring voxels, we fabricated spot arrays with different dwell times and lithography laser fluences. For this, a piezoelectric stage raster‐scanned the XY sample plane spacing voxels 10 µm apart (ΔX) over 30 µm long scan lines with inter‐line spacings of 4 µm (ΔY) (Figure 5). Voxels were hence spaced substantially further apart than the width of the excitation point spread function, with a FWHM of nominally ≃0.35 µm and experimentally ≃1 µm, as confirmed by confocal microscopy via MB autofluorescence under 571 nm excitation (Figures 2C and 5B). The previously observed 110°C excess heat plateau could be reproduced for each voxel in the array when writing with 300 nM MB and 60 mW lithography laser power and individual voxel exposures of 1.5 s, corresponding to an effective scan speed of 5 µm/s (Figure 5C). Translating the stage to a new voxel coordinate reset the recorded temperature back to ambient room temperature before the subsequent voxel polymerization returned the same characteristic temperature plateau, resulting in an overall oscillatory temperature time course (Figure 5C). Curiously, the baseline temperature increased by a modest but systematic amount of ca. 1.2°C, which we attributed to cumulative heat diffusion from previously irradiated voxels [87].

Upon doubling the effective scan speed to 10 µm/s, corresponding to a 0.75 s voxel dwell time (Figure 5D), the observed temperatures fluctuate in a similar manner together with an overall increase in temperature (Figure 5E). Measured heating maxima remained below those observed previously, which was consistent with the 50% shorter exposures. Over the course of 25 s of sequential voxel writing, the local cooling minima between two voxel exposures remained about 5°C above lab background temperature, implicating diffused heat to contribute a higher thermal load. For an increased power of 70 mW the cooling minima rose by about 13°C over time (Figure 5F), and the temperature peaks reached temperatures ≃110°C, more than twice the plateau maximum observed under 60 mW irradiation power. In addition, the temperature fluctuations became again more pronounced, similar to the 1.5 s dwell time.

Overall, the UCNP‐slide and co‐aligned lithography/ thermometry laser instrument configuration could resolve different thermal responses during fs‐DLW in real time. Higher thermometry sampling rates than the available 0.5 s frame rate would be invaluable, especially for the 0.75 s dwell times. Laser power and scan speed critically influence nonlinear reaction dynamics and thus heat accumulation and dissipation. Large 0 amplitude oscillations (>100°C) were observed at high exposure doses (Figure 5C,F), while lower doses resulted in smoother, averaged traces (Figure 5D), consistent with the nonlinear two‐photon absorption and subsequent heat dissipation. Understanding and optimizing these is essential to high‐resolution printing as well as for temperature‐sensitive applications, including biological samples.

### 2.5 Thermometry of Voxel Line Writing

**FIGURE 6:**
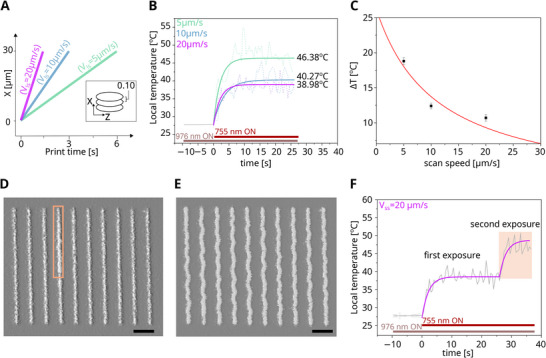
Line‐scan nanothermometry. Voxel lines were written with 60 mW lithography and imaged with 5.2 mW thermometry laser power. (A) Schematic of line‐scan experiment, indicating effective dwell‐time slopes for lines with individual voxels spaced 100 nm apart. (B) Line scan writing accumulated Δ*T* = 18.8, 12.4, and 10.7°C above lab background temperature of T = 27.8°C for scan speeds of 5, 10, and 20 µm/s respectively. (C) Temperature rise and scan speed follow a hyperbolic relation with diminishing heat accumulation at higher scan velocities. Brightfield transmission images of printed protein structures after development showed straight (D) or slightly wavy (E) voxel lines, implicating a scan speed of 20 µm/s as the underexposure limit. Scale bar = 5 µm. (F) Sequential voxel line exposures yielded ΔT = 11.0°C on the first scan and ΔT = 21.0°C on re‐exposure during a second pass. Double‐exposed line segments were noticeably thicker (D, inset).

Finally, we investigated the thermal evolution during continuous voxel line‐scans, the most prevalent mode for writing complex 3D structures via multiphoton polymerization. For this, 30 µm long lines (ΔX) were continuously scanned with an opened shutter, then the shutter was closed, and the scanner moved perpendicularly by 4 µm (ΔY), to write the next line. This procedure was adopted to minimize thermally induced polymerization in neighboring lines (Figure 6A). Continuous line scans were encoded as sequential voxel exposures spaced 100 nm apart, with overall scan speeds of 5–20 µm/s. Temperature plateaus decreased systematically with increasing scan speeds. At 300 nm MB, 60 mW lithography laser power, the temperature plateau increase was ΔT  =  18.8°C,  12.4°C, and 10.7°C for 5, 10, and 20 µm/s voxel scan speeds (Figure 6B). The writing time for a structure composed of ten lines 30 µm long, decreased in turn from 80 to 40 and 20 s, respectively. The dependence of temperature rise (Δ*T*) on scan speed (V_*ss*_) can be described by a hyperbolic relationship [88, 89]:

(9) $$\Delta T=\frac{\Delta_{0}}{1+\frac{V_{ss}}{V_{0}}}\;$$

with best‐fit parameters Δ_0_ = 26 ± 2°C and *V* = 11.9 ± 2 µm/s (Figure 6C). In the low scan speed regime of V_*ss*_ → 0, the temperature difference, Δ*T*, decreases linearly with *V_ss_* as Δ*T* ≃ Δ_0_ − *V_ss_* Δ_*o*_/ *V_o_*, as expected for a linear stage motion. For high scan speeds (V_*ss*_ → ∞) Δ*T* ≃ 0 is slowly approaching negligible values, as the stage motion is outrunning thermal diffusion. This behavior is consistent with an energy deposition model governed by [90, 91, 92]:

(10) $$H=P\;\tau_{dwell}=\frac{Pw_{0}}{V_{ss}}\;$$

where *H* is the heat delivered, *P* is the laser power, τ_*dwell*_ is the dwell time at a given location and *w*_0_  is the laser beam waist. Therefore, while temperature increase generally scales with dwell time at fixed lithography laser power, the relationship is not strictly linear due to heat diffusion, material‐specific absorption dynamics, and exothermic reaction kinetics.

In search for writing conditions that remain near physiological temperatures, we further analyzed structures written at 20 µm/s scan speed. At this speed, heat plateaus remained reliably under 39°C, which is sufficiently below the critical temperature threshold of 42–45°C for protein denaturation and ultimately cell death [25, 26]. This suggests that careful fs‐DLW parameter optimization can maintain biological integrity while producing well‐defined structures. Bright field microscopy revealed occasionally deformed line structures with some prints showing wavy edges (Figure 6D,E). These were consistent with solvent uptake induced swelling, which we attributed to be an underexposure artifact from slightly sub‐optimal defocusing in parts of the sample. Scan speeds of 20 µm/s should hence be considered near the lower polymerization threshold. We thus hypothesized that a dual exposure strategy could increase scaffold cross‐linking to reduce solvent swelling without compromising the thermal process window.

To evaluate cumulative heating effects, we conducted a double exposure experiment by rewriting the initial five lines (Figure 6D). A second exposure scan over the same line nearly doubled the thermal load, resulting in ΔT = 20.8°C, again exceeding the desired bioprocess window. Heat accumulation is hence governed not only by scan speed, but also by voxel geometry and beam placement, with double exposures amplifying structural inhomogeneities and undermining fabrication fidelity (Section S6 and Figure S4).

## 3 Conclusion

Improving protein‐based bioinks for femtosecond direct laser writing in vivo benefits from a more comprehensive understanding of the reaction process. In these systems, photoinitiated protein cross‐linking involves photochemical and photothermal mechanisms. Our results provide compelling evidence that local heat generation plays a pivotal, yet often underestimated, role in the polymerization dynamics of such materials. Thermal runaway effects in radical‐based polymerization reactions are well understood [93], but remain underexplored in femtosecond direct laser writing [5, 66, 67]. It warrants attention especially in bioprinting, where heat induced protein unfolding and cell death commonly require an effective process window below 42–45°C [25, 26]. We found voxel writing temperatures to transiently exceed 120–140°C under stationary fs‐laser exposure, without noticeable signs of excess heat, such as bubble formation above the boiling point, or heat induced damage to printed scaffolds. By operating in a sufficiently fast laser scanning regime, local heat accumulation could be reduced about tenfold to ∼10°C, which is generally considered acceptable for most in vivo or live‐cell applications [12, 18]. Systematically optimizing scan speed, laser power, as well as resin composition may help to maintain constant temperature during polymerization to help improve performance of precision microfabrication parts. This, for example, could reduce contour deformations or refractive index deviations at stitching lines in microprinted optics [3, 94, 95]. As polymerization efficiency and structural fidelity critically hinge on local thermal effects, these should hence inform future photoresist and print process engineering.

Further improved UCNP formulations and thermometry data processing will be invaluable to assess absolute temperature at high spatial and temporal resolution, which is challenging to achieve with conventional thermo‐cameras [96]. We introduced a mathematically rigorous short pass filtering to isolate optically and thermodynamically coherent transitions. In bypassing Gaussian deconvolution entirely, this approach is less prone to measurement noise and improved temperature sensitivity and accuracy of Er^3+^‐based photoluminesce nanothermometers. It is hence well suited for fast low‐intensity thermometry required for real‐time process control and optimization during femtosecond direct laser writing and multiphoton microscopy. Intriguingly, in this context, UCNPs have even been leveraged to induce photopolymerization itself [97, 98, 99, 100]. Furthermore, these efforts may help advance diverse areas aside from laser fabrication, such as microelectronics, photonics, or nanomedicine.

## 4 Materials and Methods

### 4.1 Bioink Formulation

Lyophilized bovine serum albumin (BSA, ≥96%, Sigma‐Aldrich) was dissolved in ultrapure water (Milli‐Q IQ 7000 system, MilliporeSigma) to 50–300 mg/mL final concentration and methylene blue (MB, hydrate, Sigma‐Aldrich) photo initiator, as previously reported [74]. Optimal outcomes for femtosecond direct laser writing (fs‐DLW) in this BSA‐based matrix, especially in the context of localized heating, protein denaturation, and nucleation of microbubbles, were observed across a wide range of BSA concentrations between 50–300 mg/mL. However, the concentrations of photosensitizers were carefully maintained below a critical threshold of 0.7 mM MB, beyond which uncontrolled photochemical activity led to random microbubble formation, even under moderate laser intensities. Staying below these limits and carefully choosing the polymerization laser powers preserved both the structural uniformity of the printed structures and their photothermal reliability.

Based on these considerations, we adopted a bioink recipe to minimize the risk of unwanted photothermal effects by limiting the concentration of both the BSA to 65 mg/mL and photoinitiator MB to values equal or below 0.6 mM. This highest concentration corresponds to an estimated molar ratio of six MB molecules per hundred BSA molecules, ensuring both efficient cross‐linking and minimal perturbation of the protein matrix under femtosecond laser irradiation.

### 4.2 Cell Culture

HeLa cells were cultured in Dulbecco´s Modified Eagle Medium supplemented with 10% (v/v) fetal bovine serum and 1% penicillin‐streptomycin (all EuroClone). Cells were maintained at 37°C in a humidified incubator (Galaxy 14S, New Brunswick) under 5% CO_2_ atmosphere. For live cell direct laser writing, cells were seeded into an 8‐well chambered cover glass (Ibidi) and cultured until reaching ∼70–80% confluency. Prior to fs‐DLW, culture media was gently aspirated, to then immediately add 50 µL bioink solution into each well. The sample was mounted into the inverted microscope for laser writing. Afterward, each well was carefully rinsed with phosphate‐buffered saline (PBS) to wash unpolymerized bioink away, before fresh cell culture medium was reintroduced. Cell viability was evaluated 1 and 48 h after laser writing using a live/dead staining kit (Invitrogen). Briefly, cells were incubated with calcein‐AM and propidium iodide for 15 min at 37°C, followed by confocal fluorescence imaging.

### 4.3 Temperature Sensor Film Formation

To enable real‐time thermal sensing during femtosecond direct laser writing, we employed the thermally sensitive 510–570 nm photoluminescence band of Er^3+^ ions embedded in β–NaYF_4_:(20%)Yb^3+^/(2%)Er^3+^ nanoparticles (oleic‐acid capped, 10 mg/mL in toluene, Sigma‐Aldrich; Product No. 900556). The nanoparticle‐based thermometric layer was fabricated by drop‐casting 50 µL of the stock solution onto a microscope glass slide (nominal thickness ∼170 µm). A second pre‐cleaned glass slide was placed atop and gently dragged across the first, forming a homogeneously thin nanoparticle coating on both contacting surfaces. The prepared assemblies were subsequently transferred into a nitrogen‐filled desiccator for solvent evaporation and residual toluene removal, typically for one hour (Figure 2A).

### 4.4 Optics and Instrumentation

Femtosecond direct laser writing and in situ thermometry were performed on a custom‐built two‐photon microscope designed [74] to deliver colinearly aligned fs‐DLW and thermometry excitation pump beams. Both lasers were aligned as colinear Gaussian beams and were focused to the same position inside the resist, with the smaller lithography focus nested within the broader thermometry focus. The system utilized a mode‐locked Ti:Sapphire laser (Millennia X pumped Tsunami, Newport, CA) delivering ∼200 fs pulses at an 80 MHz repetition rate. The beam was directed and tightly focused onto the sample using a dry objective lens (Nikon 60×, NA = 0.85) and delived peak intensities of ∼0.4 TW⋅cm^−2^. The thermometry pump laser, a continuous‐wave fiber‐coupled diode laser emitting at 976 nm (BL976‐PAG900, Thorlabs, Germany), was aligned colinearly with the femtosecond beam via a short pass dichroic mirror (DMSP900, Thorlabs, Germany) with a 900 nm cut‐off. This optical arrangement ensured 10 nm precise spatial overlap of the fs‐DLW and thermometry excitation beams at the sample plane. Upconversion photoluminescence was monitored in real time through a side port of the inverted microscope, relayed via a fiber‐optic bundle to a CMOS‐coupled spectrograph (Newport 77400).

### 4.5 Temperature Calibration

A Peltier stage was first calibrated by mapping applied voltage to surface temperature, measured with a calibrated thermocouple (HYP‐1, OMEGA) in direct contact with the plate and cross‐validated using an IR camera (FLIR E5). This voltage–temperature relation was then used to set defined thermal points for the UCNP thin films. At each point, emission spectra under 976 nm CW excitation were recorded to generate the calibration curve linking UCNP luminescence to absolute temperature. The same calibrated stage was subsequently used for bioink drop‐cast samples to ensure consistency across all measurements.

### 4.6 Transmission Electron Microscopy

UCNP particles were imaged on a JEOL JEM‐2100 Plus electron microscope operating at 100 kV. Samples were prepared by drop‐casting 5 µL of a 0.1 g/L UCNP stock solution onto a carbon‐coated copper grid. Nanoparticles had a narrow size distribution centered at 14 ± 0.06 nm.

### 4.7 Dynamic Light Scattering (DLS)

DLS measurements conducted on aqueous dispersions of the same nanoparticles indicated a significantly larger average diameter of approximately 30 nm (Figure S1). This twofold increase compared to DLS arises from fundamental differences between the two techniques. While TEM provides direct imaging of individual particles under vacuum, yielding high‐resolution measurements of the solid‐state core dimensions, DLS captures the hydrodynamic diameter in solution. This measurement encompasses not only the particle core but also the surrounding solvation shell, as well as any transient clusters or aggregates formed due to interparticle interactions. The observed increase in size suggests that the nanoparticles exhibit a tendency to form aggregates in aqueous environments, a behavior likely intensified by the polydispersity of the suspension and the oleic‐acid surface functionalization, which may promote interparticle affinity under certain dispersion conditions (Section S1).

### 4.8 Confocal Fluorescence Microscopy of Printed Scaffolds

Brightfield and fluorescence imaging of the printed structures were performed using a Leica TCS SP5 STED‐CW laser scanning confocal microscope (Leica Microsystems, Germany). Brightfield images were recorded using a transmitted light configuration. For fluorescence measurements, the intrinsic MB autofluorescence was excited at 571 nm using a diode‐pumped solid‐state laser (P ≈ 10 µW at the sample plane). Emission was collected through a 20×/0.5 NA air objective (HCX PL Fluotar, Leica Microsystems).

### 4.9 CIE 1931 Plot Analysis

The emission spectra of NaYF_4_:Yb^3+^/Er^3+^ thin films under 976 nm excitation at varying power levels were measured and converted into CIE 1931 chromaticity coordinates (x, y) by multiplying the intensity at each wavelength by the CIE 1931 color matching functions and calculating x = X/(X+Y+Z) and y = Y/(X+Y+Z) (Section S3).

### 4.10 Statistical Analysis

Details of spectral preprocessing, exclusion criteria, ratiometric extraction, uncertainty propagation, and calibration statistics are provided in (Section S7).

## Conflicts of Interest

The authors declare no conflicts of interest.

## Supporting information




**Supporting File**: smtd70468‐sup‐0001‐SuppMat.docx.

## Data Availability

The data that support the findings of this study are available from the corresponding author upon reasonable request.
