# Supplementary material for: Upconversion Photoluminescence to Monitor Local Heat Release During Femtosecond Direct Laser Writing of Bioinks In Situ
Source: Small Methods. 2026 Feb 8;10(4):e02130. doi: 10.1002/smtd.202502130 (PMC12929933; doi:10.1002/smtd.202502130)
Supplement: Supplementary file 1 — Supporting File: smtd70468‐sup‐0001‐SuppMat.docx. [file SMTD-10-e02130-s001.docx]

Supporting Information for

**Upconversion photoluminescence to monitor local heat release during femtosecond direct laser writing of bioinks *in situ***

*Amirbahador Zeynali^1^, Giuseppe Chirico^2^, Michael Heymann^1^*

1. *IBBS, Institut for Biomaterials and Biomolecular Systems, University of Stuttgart,
   Pfaffenwaldring 57, 70569 Stuttgart, Germany*
2. *Department of Physics, University of Milano-Bicocca, Piazza della Scienza 3, 20126, Milano, Italy*

**Section S1: Electron microscopy and dynamic light scattering to size** $\mathbf{NaY}\mathbf{F}_{\mathbf{4}}\mathbf{:}\mathbf{Yb}^{\mathbf{3+}}\mathbf{/}\mathbf{Er}^{\mathbf{3+}}$ **UCNPs**

High-resolution imaging of individual nanoparticles via transmission electron microscopy (TEM) was performed on a JEOL JEM-2100 operating at 200 kV with a nominal point resolution of $\sim0.24 nm$ (**Figure S1A**). Samples were prepared by dispersing nanoparticles in ethanol, followed by ultrasonication for 10 min to prevent agglomeration. A drop of the suspension was deposited onto a carbon-coated copper grid and allowed to dry under ambient conditions prior to imaging. UCNPs were quantified from TEM images to on average be 14 ± 0.06 nm in diameter using the Analyze Particles Fiji plugin[1,2] (**Figure S1B**).

To exclude artifacts from aggregation and undesirable solvent interactions, dynamic light scattering was used to assess the hydrodynamic diameter of nanoparticles in toluene, as a 10× dilution in water from a 2 gr/l stock in toluene. Light scattered from a vertically polarized 632.8 nm He-Ne laser with illuminating the nanoparticle dispersion was collected at a $90^{\circ}$ angle by a photomultiplier tube. Resulting intensity fluctuations were autocorrelated as[3]:

$G\left( \tau\right)= <I\left( t \right)I^{*}\left( t+\tau\right)>=G(0)e^{-Г\tau}$ (S1)

with $\Gamma$ as the decay rate of the intensity autocorrelation, related to the dynamics of the scattering particles. This is derived from the electric field correlation function:

$G_{E}\left( \tau\right)=G_{E}\left( 0 \right)e^{-DQ^{2}\tau}$ (S2)

where $D$ is the diffusion coefficient, and $Q$ is the scattering vector defined as:

$Q= \frac{4\pi n}{\lambda}\sin\frac{\theta}{2}$ (S3)

with $n$ as the refractive index of the solvent, $\lambda$ is the laser wavelength, and $\theta$ is the detection angle. Given the quadratic relation between intensity and electric field, the correlation function can be expressed as:

$G=\left| G_{E}\left( \tau\right) \right|^{2}=G(0)e^{-2DQ^{2}\tau}$ (S4)

yielding the relation:

$Г=2DQ^{2}$ (S5)

Once $D$ is obtained by fitting the measured $G\left( \tau\right)$, the hydrodynamic radius $R$ of the nanoparticles is estimated using the Stokes-Einstein equation:

$R=\frac{kT}{6\pi\eta D}$ (S6)

where $k$ is the Boltzmann constant, $T$ the absolute temperature, and $\eta$ the dynamic viscosity of the solvent. The hydrodynamic radius obtained from the fit of the auto-correlation function is $d_{DLS}=15.5\pm1.1 nm$ (**Figure S1B**). The good agreement with the TEM estimate indicates that minimal aggregation is occurring in solution .


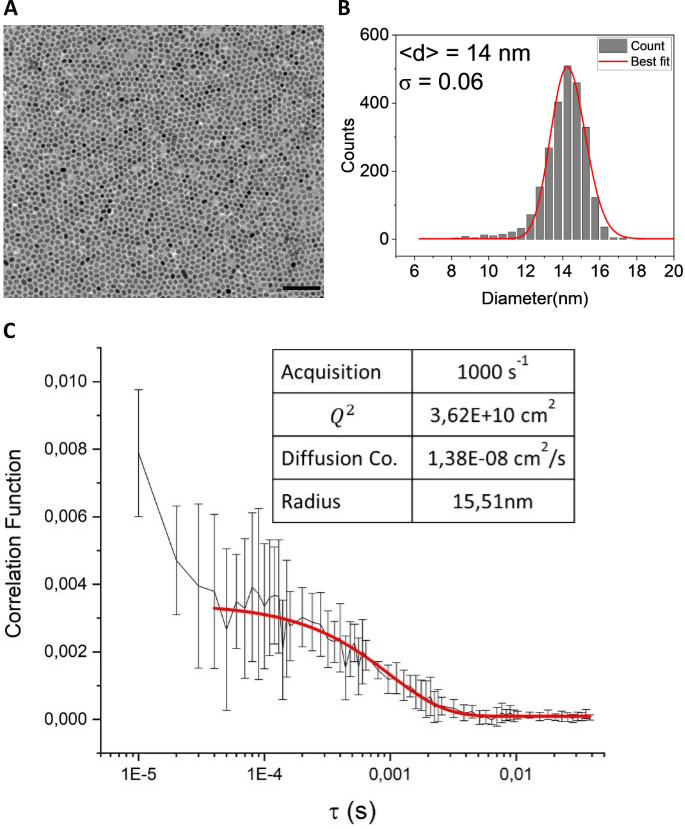


**Figure S1**. $\boldsymbol{NaY}\boldsymbol{F}_{\boldsymbol{4}}\boldsymbol{:}\boldsymbol{Yb}^{\boldsymbol{3+}}\boldsymbol{/}\boldsymbol{Er}^{\boldsymbol{3+}}$ **nanoparticle size distribution. (A)** TEM images revealed highly monodisperse **(B)** Nanoparticle sizes, with **(B)** an average diameter <d> = 14 ± 0.06 nm as obtained by TEM image analysis in Fiji. **(C)** DLS measurement and fitted correlation function (red curve) at 1kHz sampling rate for nanoparticles dispersed in water quantified a hydrodynamic of $d_{DLS}=15.5\pm1.1nm.$ Scale bar = 100 nm

**Section S2: Laser Intensity Characterization: Continuous vs. Pulsed Sources**

A continuous wave (CW) laser beam may be characterized by measuring either the output power $P$ (in Watts, W) or the intensity $I$ (in $W/cm^{2}$), defined as the power per unit cross-sectional area. In contrast, pulsed lasers deliver energy in discrete bursts, necessitating temporal averaging to correctly determine the average power $P_{avg}$ and average intensity $I_{avg}$. While these average values are helpful for comparison, they fail to capture key properties of pulsed lasers, such as pulse duration $\tau$ (in seconds) and repetition rate $f$ (in Hz). These govern peak power $P_{peak}$, peak intensity $I_{peak}$, and fluence $F$ (in $J/cm^{2}$) of the pulsed laser, which are photophysical and photochemical processes during laser-matter interaction[4]. Assuming a square-shaped pulse, these values are related through the following equations:

$P_{peak}=\frac{P_{avg}}{\tau\cdot f}$, and $I_{peak}=\frac{P_{peak}}{A}$ (S7)

where $A$ is the beam cross-sectional area at the focal plane. The area is estimated based on diffraction-limited focusing using the Airy disk model:

$A=\frac{\pi\times d^{2}}{4}$ , and $d=1.22\frac{\lambda}{N.A}$ (S8)

Here, $d$ is the beam diameter at the focus, $\lambda$ is the laser wavelength, and $N.A$ is the numerical aperture of the focusing objective.

We developed a strategy for real-time photothermal monitoring during femtosecond direct laser writing (fsDLW) using collinear CW and pulsed beams. The CW source was a 976 nm diode laser, and the femtosecond (fs) pulsed source was a Ti:Sapphire laser (755 nm) with a pulse duration $\tau\approx200$ fs and repetition rate $f=80$ MHz. Using the relation above we estimated for a 60× objective lens ($N.A$ = 0.85):

- **CW laser intensity**: $I_{avg}\approx0.3 MW/cm^{2}$
- **fs-pulsed laser peak intensity**: $I_{peak}$≈$0.4 TW/cm^{2}$

These calculations emphasize the substantial difference in peak intensities between CW and pulsed regimes and highlight the importance of accurate photothermal evaluation in laser-based micro­fab­ri­cation processes.

**Section S3: Chromaticity diagram**

The color of a luminescent material can be quantitatively described using tristimulus values ($X, Y, Z$). These are calculated from the photoluminescence spectra $S(\lambda)$ as [5]:

$X=\int_{\lambda} S\left( \lambda\right)\bar{x}\left( \lambda\right)d\lambda$ , $Y=\int_{\lambda} S\left( \lambda\right)\bar{y}\left( \lambda\right)d\lambda$ , and $Z=\int_{\lambda} S\left( \lambda\right)\bar{z}\left( \lambda\right)d\lambda$ (S9)

The corresponding chromaticity coordinates $(x,y,z)$ represent the color's hue and saturation inde­pen­dent of luminance, are derived as:

$x= \frac{X}{X+Y+Z}$ , $y= \frac{Y}{X+Y+Z}$ , and $z= \frac{Z}{X+Y+Z}$ (S10)

In Eqs. S9, $\bar{x}\left( \lambda\right)$, $\bar{y}\left( \lambda\right)$, and $\bar{z}\left( \lambda\right)$ are the CIE 1931 color matching functions for a 2° standard observer. By applying these equations to the emission spectra of the $NaYF₄:Yb^{3}⁺/Er^{3}⁺$ thin films under 976 nm excitation at varying power levels, we obtained the evolution of the chromaticity coordinates as a function of incident intensity, revealing a power-dependent shift in the ($x, y$) values (**Figure S3**).


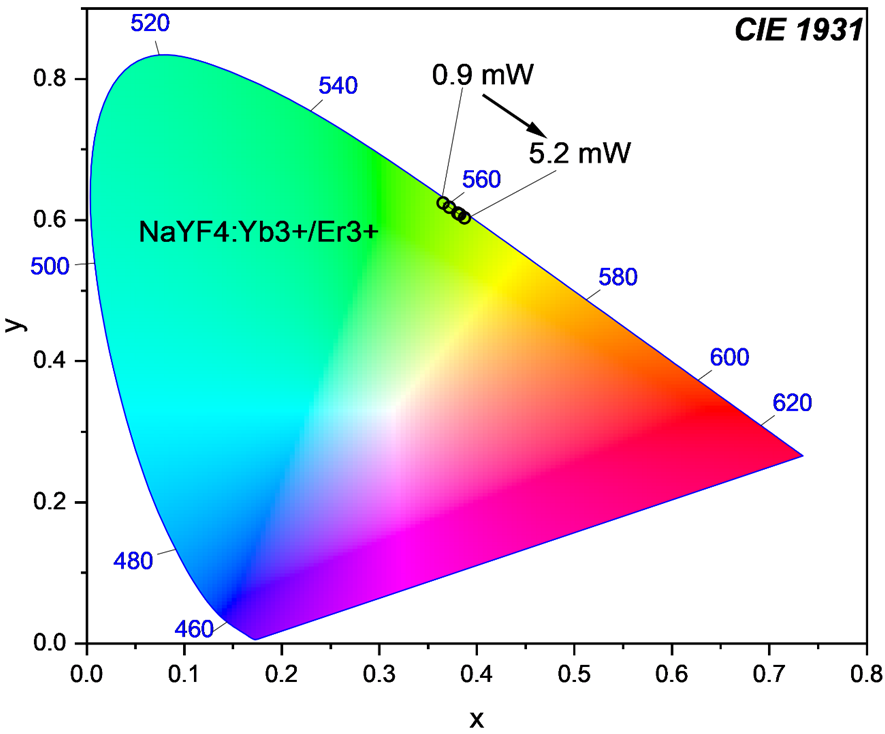


**Figure S2**. CIE colour ($x, y$) coordinates of $NaYF_{4}:{Yb}^{3+}/{Er}^{3+}$ upon increasing the 976 nm excitation laser power*.* An arrow indicates the direction of increasing power density, which correlates with a slight shift from green to yellow emission.

**Section S4: Spectral correction**

Temperature calibration of $NaYF₄:Yb^{3}⁺/Er^{3}⁺$ nanoparticle photoluminescence proceeded through advanced spectral correction to eliminate a spectral intruder band artifact emerging beyond 554 nm (**Figure S3A**). For precise Boltzmann-based calibration, we compute the intensity ratio, $\Delta$, as:

$R=\frac{I_{525}}{I_{545}}=c exp\left[ -\frac{\Delta E}{k_{B}T} \right]$, (S11)

with $I_{525}$and $I_{545}$ denoting the central emission intensities corresponding to the thermally coupled transitions ${{}^{2}H}_{11/2}\to{{}^{4}I}_{15/2}$ (centered at approximately 525 nm) and ${{}^{4}S}_{3/2}\to{{}^{4}I}_{15/2}$ (centered at approximately 545 nm), respectively. Two correction approaches were evaluated:

1) *Gaussian deconvolution (Correction 1):* employed multi-Gaussian deconvolution, by fitting the spectrum with six Gaussian components to exclude spectral components above 554 nm. Each measured luminescence spectrum $I(\lambda)$ is modeled as a sum of Gaussian functions:

$I\left( \lambda;P \right)=\sum_{i=1}^{n} G_{i}(\lambda;P)$ , (S12)

with $P$ as the excitation laser power; $n$ the number of components (here $n=6$) each defined as:

$G_{i}\left( \lambda;P \right)= A_{i}\left( P \right)\cdot exp(-\frac{\left( \lambda-\mu_{i} \right)^{2}}{2\sigma_{i}^{2}})$ , (S13)

with $A$ the amplitude of component $i$, scaling with power and the components central wavelength, *µ,* and standard deviation, $\sigma,$ related to the gaussian FWHM. The area under each gaussian component is given by:

${Area}_{i}\left( P \right)=\int_{\lambda_{1}}^{\lambda_{2}} G_{i}(\lambda;P)d\lambda$ (S14)

Empirically, the area scales with laser power as (**Figure S3 A-E**):

${Area}_{i}\left( P \right)=a_{i}P^{b_{i}}$ (S15)

By analyzing the log–log plots of the integrated area of each Gaussian component versus excitation power, we investigated the power dependence of the individual emission features (**Figure S3 F)**.

$\log\left( {Area}_{i}\left( P \right) \right)=b_{i}\log\left( P \right)+log(a_{i})$ (S16)

Components associated with the genuine photoluminescence process exhibited coherent scaling behavior, characterized by superlinear slopes (${1<b}_{i}<2$) indicative of a two-photon associated process. In contrast, two components displayed a distinctly different power-law slope, inconsistent with the behavior of the other bands, and emerged only at higher excitation powers, suggesting a multi-photon process ($b_{i}>2$). This helped to identify and isolate a non-intrinsic emission band, supporting the hypothesis of spectral contamination by a parasitic contribution.

*2) Statistical short pass filtering (Correction 2):* discarded spectral content above 554 nm via a short-pass filtering approach we developed based on the power dependence of the spectra introduced in main text; section 2.2.

The quality of both corrections was compared by linearly fitting the obtained calibration curve slopes ($\frac{\Delta E}{k_{B}T}$). *Correction 2* achieved higher resolution and reduced fitting uncertainty compared to *Correction 1*, with both corrections noticeably improving for these metrics over uncorrected spectra (**Figure S3 F**).


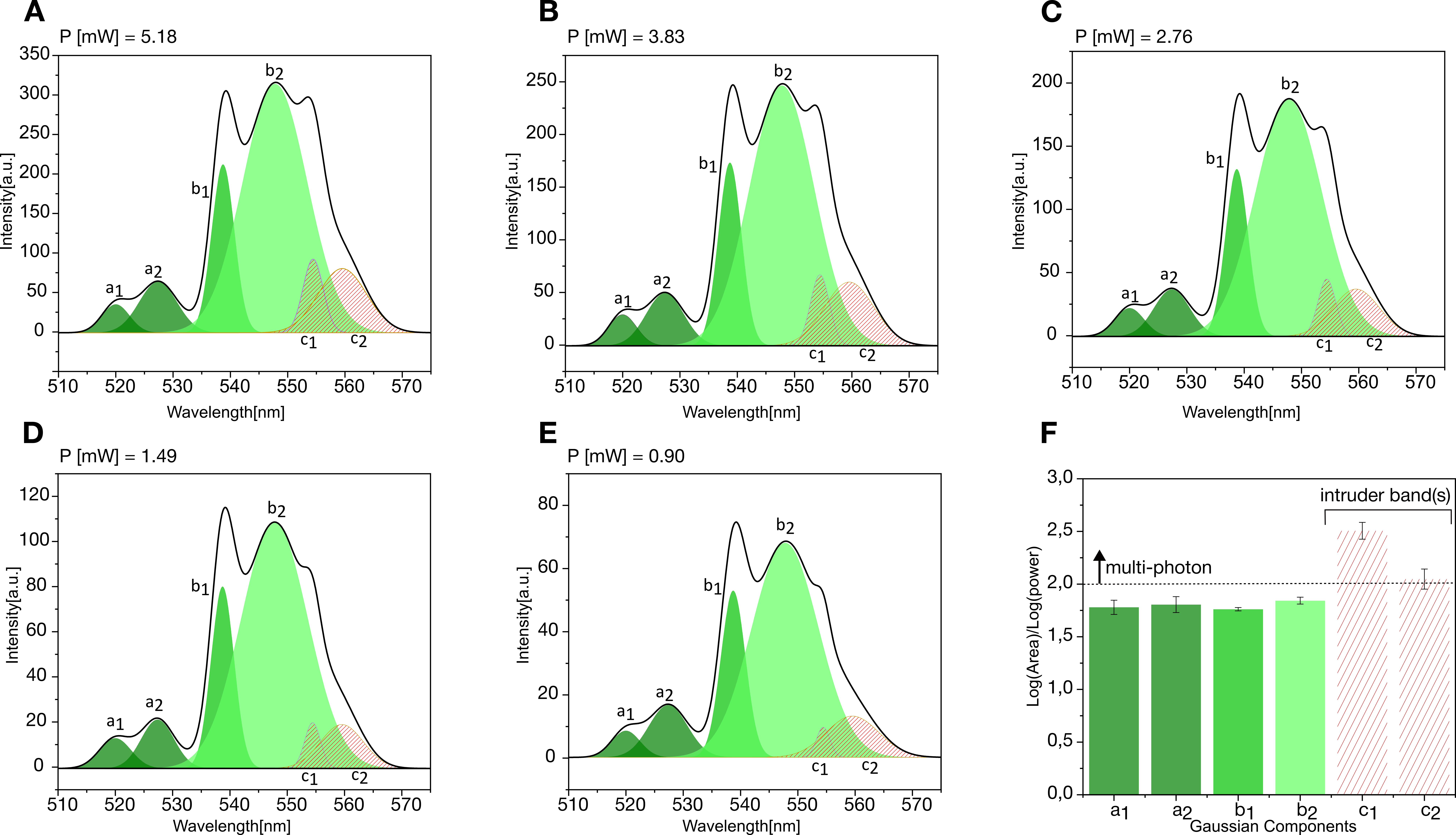


**Figure S3**. **Multigaussian deconvolution and intruder band identification. (A–E)** Deconvolution of six Gaussian components from the luminescence spectra at different excitation powers, along with the corresponding cumulative fits. **(F)** Power law exponent, derived from a linear fit over a log–log plot of integrated area versus excitation power, for each of the components identified via multi-Gaussian analysis. This analysis reveals distinct power-dependent behavior and enables identification of contaminating intruder bands shaded in red.

**Section S5: Thermally quenched emitting states,** ${{}^{\boldsymbol{2}}\boldsymbol{H}}_{\boldsymbol{11/2}}$ **and** ${{}^{\boldsymbol{4}}\boldsymbol{S}}_{\boldsymbol{3/2}}$**, energy gap (**$\boldsymbol{\Delta E}$**)**

**Table S1.** Summary of centroid wavelengths ($\lambda_{c1}$ & $\lambda_{c2}$) and energy states ($E_{1}$ & $E_{2}$) derived from the Jacobian transformation of $I(\lambda)$, used to identify band compression during temperature increases.

| **Temperature(**$\boldsymbol{^{\circ}C}$**)** | $\boldsymbol{\lambda}_{\boldsymbol{c}\boldsymbol{1}}$  $\boldsymbol{(nm)}$ | $\boldsymbol{E}_{\boldsymbol{1}}$  $\boldsymbol{(cm}^{\boldsymbol{-1}}\boldsymbol{)}$ | $\boldsymbol{\lambda}_{\boldsymbol{c}\boldsymbol{2}}$  $\boldsymbol{(nm)}$ | $\boldsymbol{E}_{\boldsymbol{1}}$  $\boldsymbol{(cm}^{\boldsymbol{-1}}\boldsymbol{)}$ | $\boldsymbol{\Delta E}$  $\boldsymbol{(cm}^{\boldsymbol{-1}}\boldsymbol{)}$ |
| --- | --- | --- | --- | --- | --- |
| **38** | 526.67 | 19023 | 547.89 | 18252 | 771 |
| **59** | 525.86 | 19016 | 547.67 | 18259 | 757 |
| **85** | 526.47 | 18994 | 547.43 | 18267 | 727 |
| **119** | 526.51 | 18993 | 547.11 | 18278 | 715 |
| **140** | 527.14 | 18970 | 546.36 | 18303 | 667 |

**Section S6: fs-DLW of parallel lines in scan-mode**


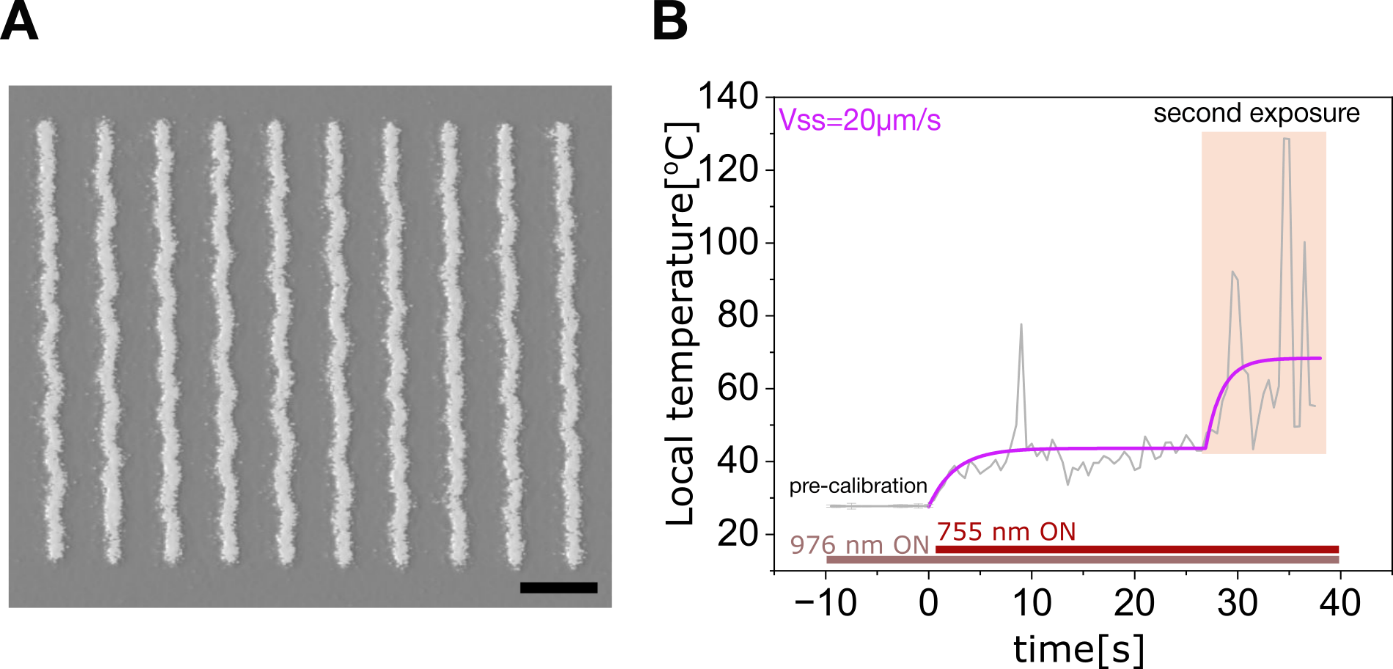


**Figure S4**. Confocal images of protein-based structures created via fsDLW using a 755 nm laser at an average power of 60 mW and a scan speed of 20 µm/s. **(A)** fsDLW conducted with the beam deeper within the bioink volume (full voxel-in), leading to thicker and less uniform polymerization and **(B)** A noisy local temperature profile with sudden heat spikes to above 100 °C. Scale bare = 5µm.

**Section S7: Statistical Analysis**

All statistical procedures and reporting follow established practices in quantitative luminescence thermometry and femtosecond direct laser writing (fs-DLW). Numerical values specific to each experiment (e.g., number of spectra, repetitions, temperature plateaus, and calibration fits) are reported in the main manuscript, figure captions, or in the corresponding supplementary sections. Statistical analyses, curve fitting, and spectral integration were performed using OriginPro (OriginLab Corporation).

*Pre-processing of raw spectra (Section S4):* All emission spectra were dark-current subtracted and wavelength-calibrated before analysis. For ratiometric thermometry, spectra were processed using (i) the conventional Gaussian deconvolution procedure (Correction Method 1) and (ii) the statistical short-pass filter (Correction Method 2) designed to suppress oversampling artifacts and remove the non-thermally responsive intruder band.

Spectral regions in which the integrated intensity exhibited a relative standard error larger than 10% were excluded from quantitative analysis to avoid propagating noise-dominated values into the thermometric ratio. All remaining steps were applied uniformly across datasets.

*Extraction of band intensities and ratio formation:* Integrated intensities were computed by numerical integration of background-corrected spectral windows corresponding to the thermally sensitive transitions:

- $\boldsymbol{I}_{\boldsymbol{525}}$: lower-energy transition (514–531 nm)
- $\boldsymbol{I}_{\boldsymbol{545}}$: higher-energy transition (534–554 nm)

Where indicated, consecutive spectra were averaged to reduce acquisition noise. No smoothing, truncation, or other automated preprocessing was applied unless stated explicitly in the figure legends.

*Propagation of uncertainty:* All reported central tendency and dispersion measures are mean ± standard deviation (SD).

Temperature is derived from the ratiometric calibration $R$ = $I_{525}$/$I_{545}$ and Boltzmann-type calibration parameters ($\Delta E$ and prefactor $c$). Uncertainty in temperature ($\delta T$) was estimated by standard Gaussian error propagation from the uncertainties in the directly measured and fitted quantities. For the calibration form

$T=\frac{\Delta E}{k_{B}ln(\frac{c}{R})}$ (S17)

We used

$\delta T=\sqrt{{(\frac{\partial T}{\partial R}\delta R)}^{2}+{(\frac{\partial T}{\partial\Delta E}\delta\Delta E)}^{2}+\sum{(\frac{\partial T}{\partial p_{i}}\delta p_{i})}^{2}}$ (S18)

where $p_{i}$ accounts for other calibration parameters and the partial derivatives as follow:

$\frac{\partial T}{\partial R}=\frac{\Delta E}{k_{B}R\cdot\ln^{2}(\frac{c}{R})} , \frac{\partial T}{\partial\Delta E}=\frac{1}{k_{B}\cdot ln(\frac{c}{R})} , \frac{\partial T}{\partial c}=\frac{-\Delta E}{k_{B}c\cdot\ln^{2}(\frac{c}{R})}$ (S19)

The uncertainty in the intensity ratio was calculated assuming independent spectral-integration errors as:

$\delta R=\sqrt{{(\frac{\delta I_{525}}{I_{525}})}^{2}+{(\frac{\delta I_{545}}{I_{545}})}^{2}}$ (S20)

and calibration parameter uncertainties ($\delta\Delta E$, $\delta c$) were taken from the covariance matrix of the calibration fit. Typical propagated temperature uncertainties after filtering are reported in Table 2 ($\delta T \approx0.2-0.4 K$).

In the general case, the uncertainty in temperature was estimated by full Gaussian error propagation using the partial derivatives of the calibration function. However, once the calibration parameters ($\Delta E$ and prefactor $c$) are fixed, their contribution to temperature uncertainty becomes negligible relative to the measurement uncertainty in the intensity ratio $R$. Under this experimentally valid condition, the expression reduces to a widely used first-order approximation in luminescence thermometry:

$\delta T=\frac{1}{S_{r}}\frac{\delta R}{R}$ (S20)

**References**

1. Schindelin J, Arganda-Carreras I, Frise E, et al. Fiji: an open-source platform for biological-image analysis. *Nat Methods*. 2012;9(7):676-682. doi:10.1038/nmeth.2019

2. Sankur B. Survey over image thresholding techniques and quantitative performance evaluation. *J Electron Imaging*. 2004;13(1):146. doi:10.1117/1.1631315

3. Pine DJ, Weitz DA, Zhu JX, Herbolzheimer E. Diffusing-wave spectroscopy: dynamic light scattering in the multiple scattering limit. *Journal de Physique*. 1990;51(18):2101-2127. doi:10.1051/jphys:0199000510180210100

4. Bouzin M, Zeynali A, Marini M, et al. Multiphoton Laser Fabrication of Hybrid Photo-Activable Biomaterials. *Sensors*. 2021;21(17):5891. doi:10.3390/s21175891

5. Mortimer RJ, Varley TS. Quantification of colour stimuli through the calculation of CIE chromaticity coordinates and luminance data for application to in situ colorimetry studies of electrochromic materials. *Displays*. 2011;32(1):35-44. doi:10.1016/j.displa.2010.10.001
